# Neo-generation of neogenetic bullae after surgery for spontaneous pneumothorax in young adults: a prospective study

**DOI:** 10.1186/s13019-019-0848-4

**Published:** 2019-01-23

**Authors:** Takuya Onuki, Tomoyuki Kawamura, Shuntaro Kawabata, Masatoshi Yamaoka, Masaharu Inagaki

**Affiliations:** Department of General Thoracic Surgery, Tsuchiura Kyodo General Hospital, 4-1 Ohtsuno, Tsuchiura, Ibaraki, 300-0028 Japan

**Keywords:** Spontaneous pneumothorax, Video-assisted thoracic surgery, VATS, Bullectomy, Neogenetic bullae (blebs)

## Abstract

**Background:**

To investigate the development of neogenetic bullae or blebs on 1-year postoperative chest computed tomography after video-assisted thoracic surgery (VATS) in young patients with primary spontaneous pneumothorax (PSP).

**Methods:**

In this prospective study, 10- to 20-year-old patients with PSP were treated via VATS with additional procedures (bullectomy, cold coagulation, coverage, pleural abrasion, or chemical pleurodesis). All patients underwent the additional procedures and computed tomography of the chest 1 year postoperatively for the assessment of neogenetic bullae. Postoperative PSP recurrence was monitored, and recurrence-free survival was evaluated using Kaplan-Meier analysis.

**Results:**

Fifty-seven patients (66 cases) aged 17 ± 2 years underwent VATS for PSP and were followed up for 938 ± 496 days. Of the 36 cases at 1-year follow-up, 23 (63.9%) showed neogenetic bullae, which were adjacent to the staple lines in 16 cases (69.6%). The 1- and 2-year recurrence-free survival rates were 88.9 and 85.1%, respectively. Nine of the 66 cases (13.6%) showed recurrence after 869 ± 542 days. A history of contralateral PSP was significantly associated with recurrence.

**Conclusions:**

VATS, combined with additional procedures, provides acceptable long-term results in young patients with PSP. Additional procedures reduce the recurrence rate of PSP but do not prevent the occurrence of neogenetic bullae. A history of contralateral PSP is a potential risk factor for post-VATS recurrence in young patients.

## Background

Pneumothorax is a respiratory disorder involving abnormal air accumulation in the thoracic cavity and is classified as spontaneous, traumatic, or iatrogenic [1]. Spontaneous pneumothorax (SP) occurs without any trauma or obvious precipitating factors and includes primary spontaneous pneumothorax (PSP), which occurs in healthy people, and secondary spontaneous pneumothorax (SSP), which occurs in people with preexisting lung diseases [1, 2]. The annual incidence of PSP among men and women ranges from 7.4 to 28 (age-adjusted incidence) and 1.2 to 6 cases per 100,000 population, respectively. PSP is known to be particularly common in young men with a tall and thin body shape [3].

A small SP is frequently asymptomatic and conservatively managed by rest and observation. A significant accumulation of air in the thoracic cavity requires management by needle aspiration, chest drainage, or surgical intervention [2]. Common surgical indications for PSP include a second ipsilateral pneumothorax, first contralateral pneumothorax, simultaneous bilateral pneumothorax, and persistent air leakage [2]. A bullectomy using video-assisted thoracic surgery (VATS) is currently the preferred surgical treatment for SP owing to its minimally invasive nature [4]. However, VATS is associated with a higher postoperative recurrence rate than open surgery [5], particularly in younger patients [6–8].

The development of neogenetic bullae is thought to be one of the most important causes of postoperative recurrence [5–10]. In addition to bullectomy using an endoscopic linear stapler, surgical procedures such as partial pulmonary resection, coagulation of the staple lines, coverage of the staple lines and adjacent areas with polyglycolic acid (PGA) sheets and fibrin glue, apical pleural abrasion, and chemical pleurodesis are often performed to prevent the development of neogenetic bullae and the recurrence of pneumothorax [11–20].

However, previous studies have not examined the postoperative chest computed tomography (CT) images beyond 1–2 years after surgery and our understanding of the role of neogenetic bullae in long-term recurrence is limited. We hypothesize that VATS together with additional procedures such as bullectomy would prevent neogenetic bullae and a recurrence of pneumothorax. Here, we investigated the development of neogenetic bullae on the basis of 1-year postoperative chest CT images and their relationship with postoperative PSP recurrence following VATS in young patients with PSP in a prospective clinical study.

## Methods

### Patient enrollment

Patients between the ages of 10 and 20 years who underwent surgery for PSP at the Tsuchiura Kyodo General Hospital, Ibaraki, Japan between January 2013 and December 2016 were enrolled in this prospective study. None of the patients had a history of lung disease, and patients with SSP, traumatic, or iatrogenic pneumothorax were excluded. The study was approved by the institutional review board of the Tsuchiura Kyodo General Hospital (approval Nos. 534 and 623). All the patients and their parents provided informed consent.

### Surgical procedure for spontaneous pneumothorax

All patients received standardized treatment using VATS. The bullae or blebs causing pneumothorax were resected using an endoscopic linear stapler (ECHELON®; Johnson & Johnson, Tokyo, Japan) and treated in the same manner. An approximately 5-mm area around each bulla was resected using the stapler. When the bullae were too small to be resected, the bullae and the surrounding region around the staple line, where micro-bullae could potentially exist, were coagulated using an ENDO-FB® (Medtronic, Tokyo, Japan) or the monopolar soft coagulation mode of the VIO300D® (ERBE, Tübingen, Germany). Next, the staple lines and adjacent areas were covered using a PGA sheet (NEOVEIL®; Gunze, Tokyo, Japan) and coated with fibrin glue (Beriplast®; CSL Behring, Tokyo, Japan). A pleural abrasion was also performed on the top of the thoracic cavities. The chest drain was removed on postoperative day 2, and 200 mg minocycline was concurrently injected into the thoracic cavity to achieve chemical pleurodesis.

### Follow-up

All patients were followed up at the hospital’s ambulatory practice for 3 months and advised to revisit the hospital for a 1-year follow-up and chest CT. The patients were also advised to visit the hospital or their family physician if they experienced chest pain or dyspnea. At their 1-year follow-up, the patients were asked about PSP recurrence (diagnosed at the study site or another hospital) and evaluated using a non-contrast chest CT scan. Patients who did not revisit the hospital for their 1-year follow-up and chest CT were followed up via mail to obtain data about their long-term results.

### Postoperative recurrence of PSP

Postoperative recurrence was defined as the development of an ipsilateral pneumothorax after postoperative day 31 (ipsilateral pulmonary collapse within the first postoperative month could be a result of delayed healing) diagnosed by clinicians using chest radiography or CT. Cases in which diagnostic imaging was not performed were not diagnosed with PSP recurrence. The observation period (from the day of surgery to the last follow-up) and the recurrence-free period (from the day of surgery to the day of postoperative PSP recurrence diagnosis) were recorded for all cases.

### Statistical analysis

Data are expressed as means and standard deviations (SD). Patient demographic variables were compared between cases with and without postoperative PSP recurrence using the chi-square test, Fisher exact test, or Student *t*-test, as appropriate. The long-term results of postoperative recurrence-free survival were evaluated using the Kaplan-Meier method. Recurrent curves were evaluated with the log-rank test. The SPSS software, version 22 (IBM Japan, Tokyo, Japan) was used for the statistical analyses. Differences were considered significant at *p*-values of < 0.05.

## Results

### Patient characteristics

A total of 57 patients underwent surgery for 66 instances of PSP at the study site between January 2013 and December 2016 (Table 1). Nine patients received VATS for bilateral PSP, of whom two had simultaneous bilateral PSP and underwent simultaneous bilateral surgery. The patients’ mean age and body mass index (BMI) calculated using data from all 66 cases at the time of surgery were 17 ± 2 years and 19.0 ± 1.8 kg/m^2^, respectively. The patients had a mean hospital stay of 4 days (median 4 days, range 4–6 days) and an observation period of 938 ± 496 days (median 939 days, range 21–1766 days). No major complications occurred during the perioperative or follow-up period.

**Table 1:** Characteristics of patients with pneumothorax

	Total	Presented for 1-year follow-up	Did not present for 1-year follow-up	*P* -value
Number of cases with pneumothorax	66	36	30	–
Number of patients	57	30	27	–
Male/female (cases)	60/6	33/3	27/3	0.81
Left/right (cases)	38/28	22/14	16/14	0.52
Age (years)				
Range	14–20	14–20	15–20	0.03
Mean ± SD	17 ± 2	17 ± 2	18 ± 1	
Median	17	17	18	
Height (cm)				
Range	156.5–184.5	156.5–183.0	158.0–184.5	0.53
Mean ± SD	171.8 ± 6.5	172.0 ± 6.5	171.2 ± 6.7	
Median	173.8	174.0	172.3	
Weight (kg)				
Range	42.0–72.5	42.0–70.9	43.0–72.5	0.99
Mean ± SD	56.0 ± 7.2	56.3 ± 7.3	55.6 ± 7.2	
Median	55.3	56.4	54.3	
Body mass index (kg/m^2^)				
Range	14.6–23.8	15.1–23.8	14.6–22.2	0.68
Mean ± SD	19.0 ± 1.8	19.1 ± 1.9	18.9 ± 1.8	
Median	19.0	18.9	19.0	

*SD* standard deviation

A total of 30 patients accounting for 36 (54.5%) of the 66 cases, who underwent VATS for PSP, presented for the 1-year follow-up (Table 1). A further 24 patients (27 PSP cases) were followed up by mail. Three cases were lost to follow-up.

### One-year CT

**Fig. 1:**
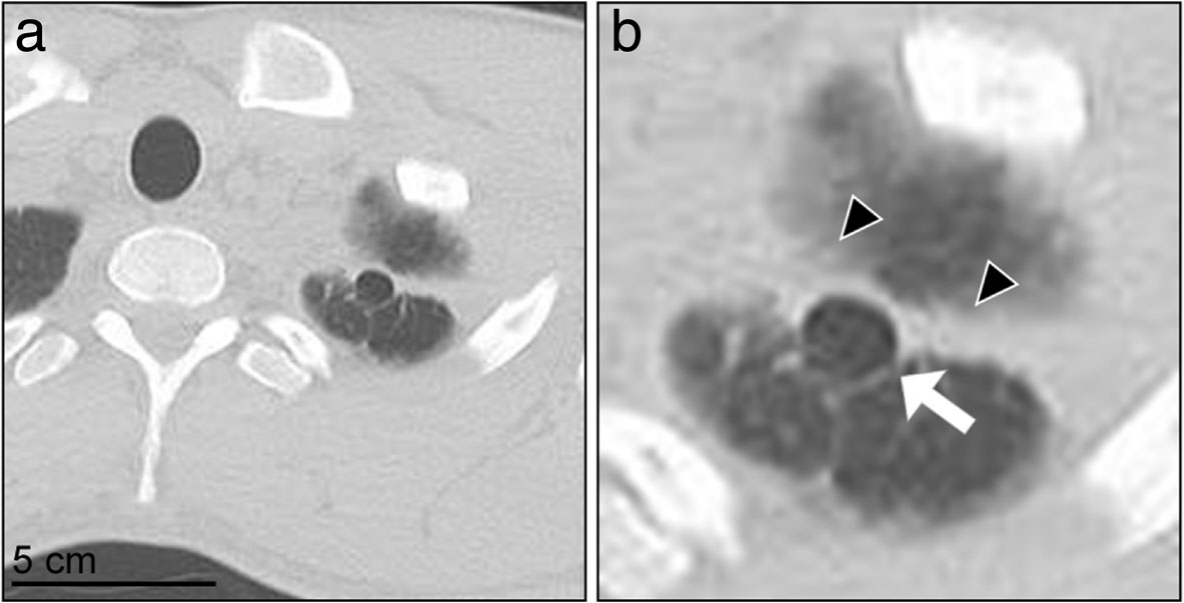
One-year chest computed tomography scan showing typical neogenetic bullae adjacent to the staple line. **a** Lower magnification of the left apex; **b** Higher magnification of the left apex. The white arrow indicates the neogenetic bullae, while the black arrowheads indicate the staple lines

**Fig. 2:**
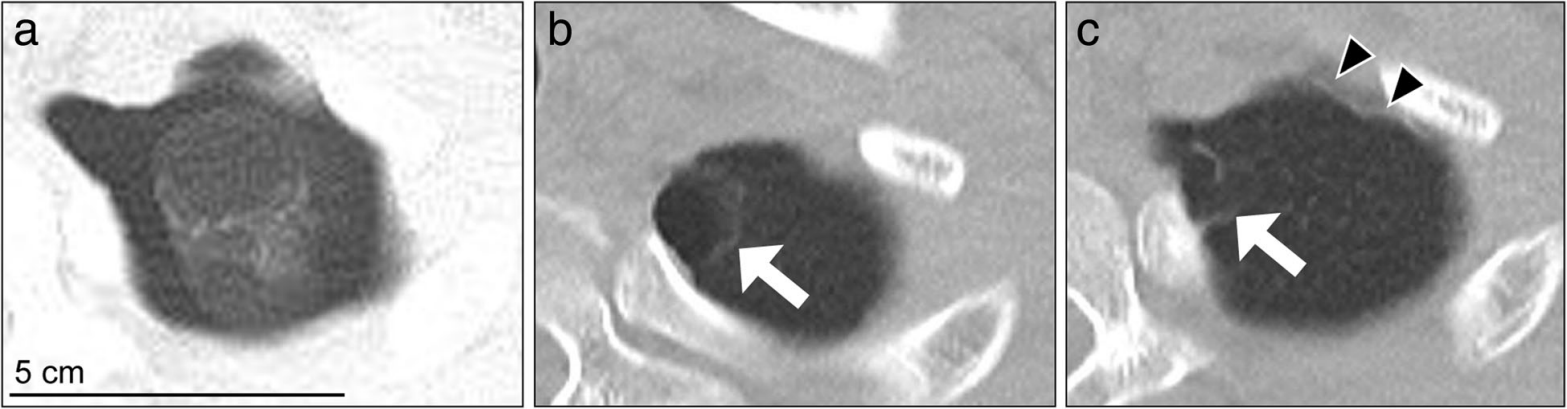
Chest computed tomography scan showing typical neogenetic bullae neighboring the staple line. **a** Preoperative findings of the pulmonary apex: the left side preoperative pneumothorax can be seen, but the bullae on the apex that are detected postoperatively are not visible. **b** One-year postoperative findings clearly show bullae on the apex. **c** At a higher magnification, the staple line can be seen. The white arrow indicates the neogenetic bullae, while the black arrowheads indicate the staple lines

The characteristics of the 36 cases followed up with a 1-year CT are presented in Table 1. Of the patients, four had a recurrence before the 1-year CT, and another four had a recurrence after the 1-year CT. No statistically significant differences in demographic variables, except for age, were found among the 36 cases followed up and the 30 cases that were not followed up, but the difference (17 ± 2 vs. 18 ± 1 years) was not clinically significant. Of the 36 cases, 23 (63.9%) showed postoperative neogenetic bullae or blebs (Table 2). Neogenetic bullae were observed on the ipsilateral upper lobe, and not in the middle or lower lobes. The neogenetic bullae were located along the staple lines for bullectomy; 16 (69.6%) of the 23 cases had neogenetic bullae “adjacent to” (i.e., within 1 cm) of the staple lines, including three cases that also had bullae on segment 6 of the lower lobe. Seven (30.4%) of 23 cases had neogenetic bullae on the upper lobe “neighboring,” (i.e., between 1 and 3 cm) of the staple lines. Figures 1 and 2 demonstrate the typical appearance of neogenetic bullae “adjacent to” and “neighboring” the staple lines.

**Table 2:** Neogenetic pulmonary bullae on 1-year computed tomography (*n* = 36)

	Number of cases
Neogenetic bullae	23 (63.9%)
Adjacent to the staple line	16
Neighboring the staple line	7
No neogenetic bullae	13 (36.1%)

### Postoperative PSP recurrence

Recurrence was observed in nine cases (13.6%) of PSP after a recurrence-free period of 869 ± 542 days (median 868 days, range 21–1766 days). The demographic and intraoperative details of these nine cases are presented in Table 3. For eight of the nine cases with PSP recurrence, 1-year CT scan was performed, and seven cases showed the presence of neogenetic bullae. Six of the nine cases were mild and managed by observation only, while the other three were managed by continuous chest drainage followed by chemical pleurodesis. The chemicals used in pleurodesis were either minocycline or a combination of talc and minocycline. None of the patients underwent a second operation for postoperative PSP recurrence. One patient received bilateral surgery and developed bilateral recurrences (cases 1 and 7; Table 3). Second recurrences were observed in two patients (cases 5 and 9; Table 3); both were mild and were managed with observation.

**Table 3:** Patients with postoperative PSP recurrence

Case number	Sex	Age (years)	Laterality	Neogenetic bullae on 1-year CT	Period from surgery to first Rec (days)	Therapy for Rec	
						First Rec	Second Rec
1	M	16	L	+	38	Observation only	–
2	M	16	L	+	36	Observation only	–
3	M	16	L	+	280	Observation only	–
4	M	17	L	+	224	Observation only	–
5	M	18	R	NA	48	Drainage and pleurodesis	Observation only
6	M	18	R	+	628	Drainage and pleurodesis	–
7	M	18	R	+	60	Drainage and pleurodesis	–
8	M	18	L	+	34	Observation only	–
9	M	20	L	–	419	Observation only	Observation only

*CT* computed tomography, *PSP* primary spontaneous pneumothorax, *Rec* recurrence, *M* male, *L* left, *R* right; drainage, continuous chest drainage

NA: Case 5 did not have 1-year CT/follow-up

**Fig. 3:**
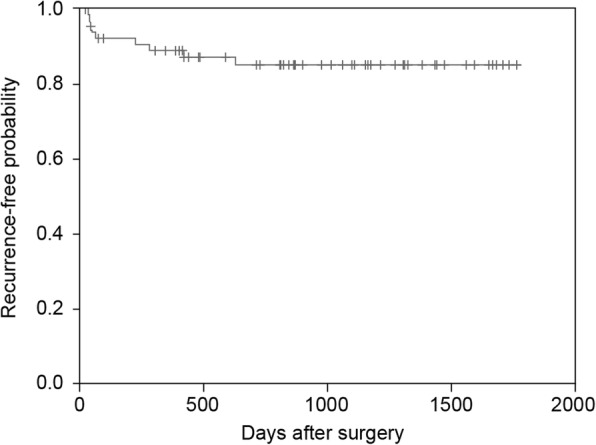
Recurrence-free survival after video-assisted thoracic surgery for spontaneous pneumothorax

The Kaplan-Meier analysis showed that 1- and 2-year recurrence-free survival rates were 88.9 and 85.1%, respectively (Fig. 3). The first recurrence occurred within 2 years after surgery in all nine cases of recurrent PSP.

The comparison between recurrent and non-recurrent cases showed no significant differences in age, sex, height, weight, BMI, or laterality (Table 4). However, recurrent cases had a significantly higher frequency of a history of contralateral PSP, including synchronous bilateral PSP, than non-recurrent cases (seven of nine recurrent cases vs. 19 of 57 non-recurrent cases, *p* = 0.01; Table 4).

**Table 4:** Comparison of epidemiological factors between patients with and patients without postoperative PSP recurrence

	Recurrence	Non-recurrence	*P*-value
Number of patients	9	57	
Age, years (mean ± SD)	17 ± 1.6	17 ± 1.3	0.78
Sex (male/female)	9/0	51/6	0.31
Height, cm (mean ± SD)	174.2 ± 6.4	171.4 ± 6.5	0.23
Weight, kg (mean ± SD)	56.7 ± 7.0	56.0 ± 7.3	0.89
Body mass index (mean ± SD)	18.6 ± 2.3	19.1 ± 1.8	0.41
Laterality (left/right)	6/3	32/25	0.55
History of contralateral PSP (yes/no)	7/2	19/38	0.01

*PSP* primary spontaneous pneumothorax

## Discussion

In this prospective study, VATS with additional procedures (resection, stapling, coagulation, coverage with PGA sheet and fibrin glue, pleural abrasion, or chemical pleurodesis using minocycline) resulted in acceptable long-term results in young patients with PSP. We performed pleurodesis by minocycline on postoperative day 2. We specifically adopted this method at our institute to prevent spontaneous pneumothorax recurrence. No major perioperative or postoperative complications occurred. Postoperative PSP recurrence developed in 13.6% of the cases after a recurrence-free period of 869 ± 542 days, while the 1- and 2-year recurrence-free survival rates were 88.9 and 85.1%, respectively. These results are in agreement with previous studies that reported recurrence rates ranging from 10.9 to 27.9% and 1-year recurrence-free survival rates ranging from 88 to 90% following VATS in young patients with PSP [6–8, 15]. Compared to open surgery, VATS for spontaneous pneumothorax has previously been reported to result in a four-fold higher recurrence rate [5]. Our patients might also have achieved better long-term results if they had received open surgery. However, VATS has been approved for spontaneous pneumothorax because of its minimal invasiveness and the shorter hospital stays associated with it [5].

Comparison between recurrent and non-recurrent cases revealed that a history of contralateral PSP (i.e., patients with bilateral PSP) was associated with postoperative PSP recurrence. Thus, a history of contralateral PSP could be considered an epidemiological risk factor to predict postoperative PSP recurrence following VATS in young patients. Such patients may have underlying genetic or environmental factors that predispose them to the development of bilateral PSP, neogenetic bullae, and recurrence. It should also be noted that the neogenetic bullae developed on the upper lobe of the bullectomy sites and were frequently (69.6%) adjacent to the staple lines. These results suggest that the staple lines directly influenced postoperative neogenetic bulla formation. We suspect that the abnormal intrapleural pressure distribution along the staple lines, together with the weakening of the pleura from tensional forces due to the use of autosutures and staples [21], could predispose patients to the formation of neogenetic bullae in the adjacent tissues. However, we were unable to find any studies describing similar observations.

The present study also presents some interesting insights into the relationship between postoperative neogenetic bullae and PSP recurrence. It is known that neogenetic bullae commonly develop following bullectomy [6, 10, 22], and it has been postulated that these neogenetic bullae contribute significantly to postoperative recurrence [6, 10, 21]. Our prospective study followed up patients for 938 ± 496 days and showed that in contrast to the 63.9% of cases with neogenetic bullae at 1 year (404 ± 101 days), only 13.6% of cases developed a recurrence in 869 ± 542 days. These results suggested that additional procedures performed with VATS do not necessarily prevent the development of neogenetic bullae and early phase recurrence, such as recurrence after one month, but they do reduce the rate of rupture of these bullae and postoperative PSP recurrence [11–20]. Our results also suggest that the additional procedures likely result in milder postoperative recurrences, as most cases with recurrence were managed conservatively and none required surgical intervention.

The additional procedures (coverage with PGA sheet and fibrin glue, pleural abrasion, and chemical pleurodesis using minocycline) performed with VATS were not associated with any major perioperative or postoperative complications in this study. We performed pleural abrasion gently and focally on the pleural apex to prevent complications such as inoperative bleeding and postoperative drainage that were previously associated with mechanical pleurodesis [23]. We also used minocycline instead of talc for chemical pleurodesis in this study because of concerns regarding the use of talc in young patients with PSP [12, 24, 25] and since minocycline has been shown to be a safe option [26, 27]. Thoracic surgeons may have experienced that administration of intrapleural minocycline may result in adhesion of the thoracic wall and lung, making future thoracic surgeries difficult. However, in our experience, this adhesion is not very strong, and it is still possible to perform future procedures if required. Indeed, the use of minocycline in our technique resulted in good long-term results and with minimal recurrence. In addition, none of the patients in our study reported or complained of dyspnea during exercise, indicating normal respiratory function. Furthermore, we also incorporated fibrin glue for staple line coverage to improve sealing of the staple lines [7, 16] and to reduce postoperative PSP recurrence. The lack of complications suggested the safety of minocycline and fibrin glue in young patients with PSP. Currently, the use of these additional drugs and techniques are not the global standard; however, they may be considered in the future as the standard of care.

Our study has some limitations. First, it was difficult to determine the time of postoperative recurrence. In the case of partial pneumonectomy, respiration results in unnatural pressure along the resection line. Owing to this pressure, the visceral pleura (pulmonary surface) near the staple line becomes slightly unstable following surgery while the staple line is still healing. Hence, wound healing requires a certain amount of time. We defined “postoperative SP recurrence” as recurrence developing more than 31 days (a month) after surgery. Perioperative re-collapse was observed in one patient within 30 days. The lack of a standard definition for postoperative SP recurrence limited our ability to compare recurrence rates with those of other studies directly. For cases with recurrence after 2 months following surgery, pulmonary wound healing (stabilization) at the staple line was considered to be incomplete. This indicated that such cases require more than 1 month to achieve wound healing. Details of surgical procedures vary depending on the medical institution; early recurrence could be influenced by the surgical technique used. Hence, it is difficult to determine the onset of postoperative recurrence, and the definition of postoperative recurrence of pneumothorax needs be clearly described. Second, this was a single-arm prospective study that did not include cases treated with bullectomy only. Thus, prospective randomized long-term studies that compare “bullectomy only” and “bullectomy with additional procedures” groups will help improve our understanding of the role played by these additional procedures in the development of neogenetic bullae and PS recurrence.

## Conclusion

VATS-based bullectomy and additional procedures such as cold coagulation, coverage of the staple line, pleural abrasion, and chemical pleurodesis resulted in acceptable long-term results in young patients with PSP. While the additional procedures did not eliminate the formation of neogenetic bullae, they likely inhibited their rupture and were associated with low recurrence rates of PS. A history of contralateral PSP might represent a risk factor for predicting postoperative PSP recurrence following VATS in young patients.

## Abbreviations

CT: Chest computed tomography
PGA: Polyglycolic acid
PSP: Primary spontaneous pneumothorax
SD: Standard deviation
SP: Spontaneous pneumothorax
SSP: Secondary spontaneous pneumothorax
VATS: Video-assisted thoracic surgery
